# Supplementary material for: Generating dual structurally and functionally skin-mimicking hydrogels by crosslinking cell-membrane compartments
Source: Nat Commun. 2024 Jan 27;15:802. doi: 10.1038/s41467-024-45006-7 (PMC10821872; doi:10.1038/s41467-024-45006-7)
Supplement: Supplementary file 1 — Supplementary Information [file 41467_2024_45006_MOESM1_ESM.pdf]

Supplementary Information

Generating dual structurally and functionally skin-mimicking hydrogels by crosslinking cell-membrane compartments

Feng Wu[§,†,#], Yusheng Ren[§,†,#], Wenyan Lv[†,⊥], Xiaobing Liu[†,⊥], Xinyue Wang[†], Chuhan Wang[‡], Zhenping Cao[†], Jinyao Liu[†,*], Jie Wei[§,*], Yan Pang[‡,*]

[§]Shanghai Key Laboratory of Advanced Polymeric Materials, School of Materials Science and Engineering, East China University of Science and Technology, Shanghai, 200237, China.

[†]State Key Laboratory of Systems Medicine for Cancer, Shanghai Cancer Institute, Shanghai Key Laboratory for Nucleic Acid Chemistry and Nanomedicine, Institute of Molecular Medicine, Renji Hospital, School of Medicine, Shanghai Jiao Tong University, Shanghai 200127, China.

[‡]Shanghai Key Laboratory of Orbital Diseases and Ocular Oncology, Department of Ophthalmology, Shanghai Ninth People's Hospital, School of Medicine, Shanghai Jiao Tong University, Shanghai 200011, China.

[⊥]College of Chemistry and Materials Science, Shanghai Normal University, Shanghai 200234, China

[#]These authors contributed equally to this work.

[*]Correspondence: Y.P. (yanpang@sjtu.edu.cn); J.W. (jiewei7860@sina.com); J.L. (jyliu@sjtu.edu.cn)

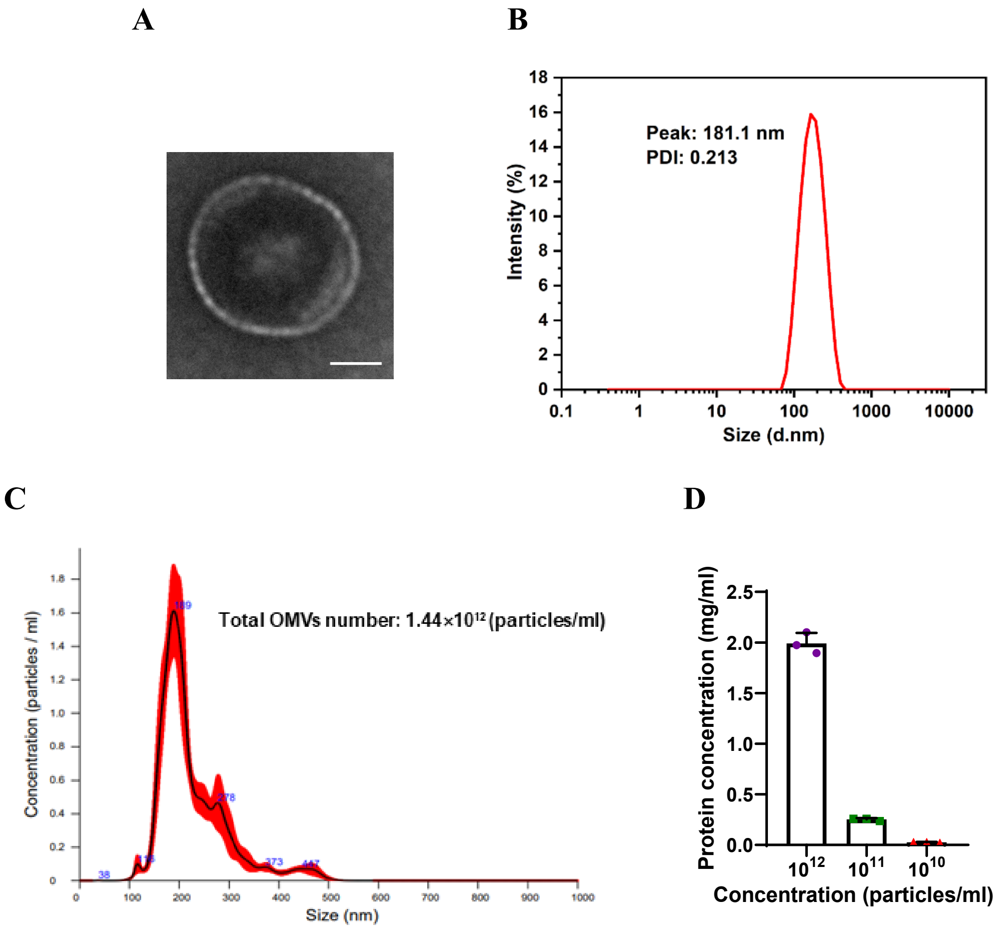

Supplementary Figure 1 (**A**) A representative TEM image of OMVs. Scale bar: 50 nm. (**B**) Size distribution of OMVs measured by DLS. (**C**) NTA curve of concentrated OMVs. (**D**) Total protein concentration of OMVs at different particle numbers obtained by BCA protein quantification kit. Data are presented as mean values ± SD (n = 3, from independent experiments).

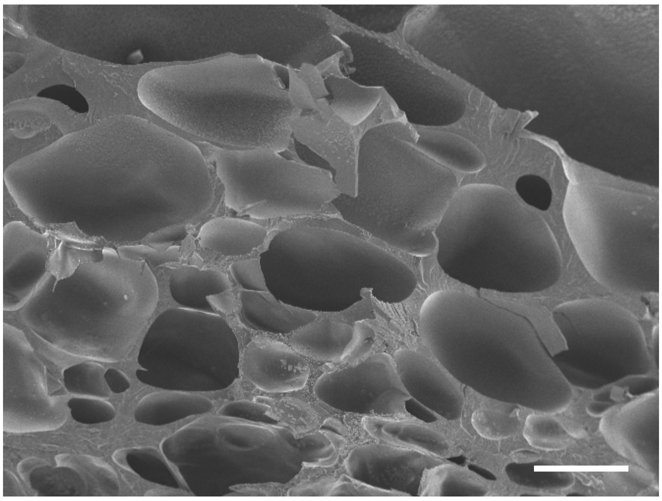

Supplementary Figure 2 A typical SEM image of lyophilized control polyacrylamide hydrogel. Magnification: × 90. Scale bar: 100 μm.

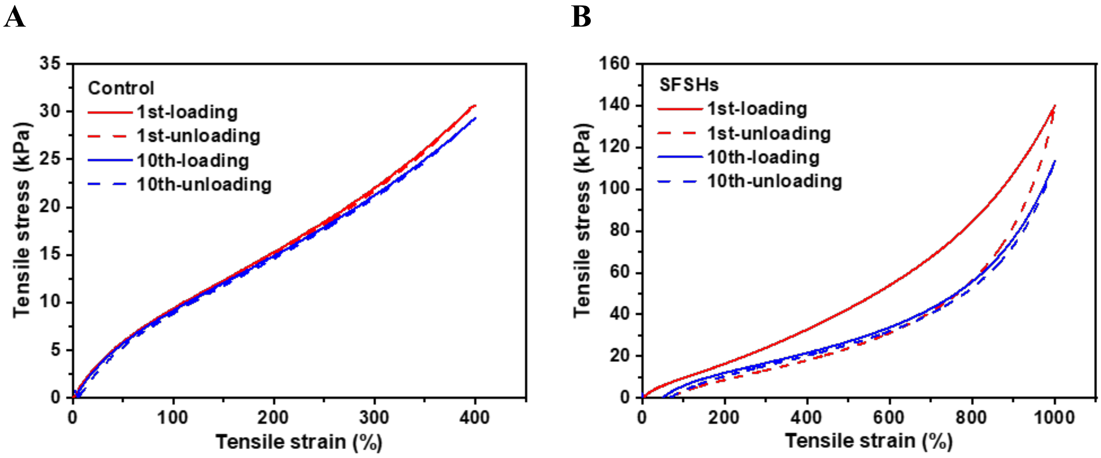

Supplementary Figure 3 Cyclic tensile of (**A**) control polyacrylamide hydrogel under 400% tensile strain and (**B**) SFSHs under 1000% tensile strain.

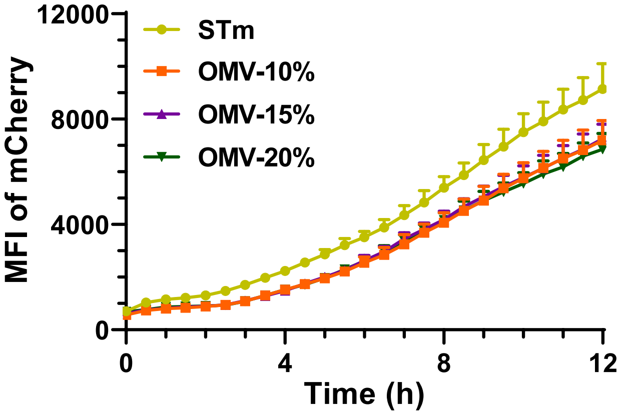

Supplementary Figure 4 Survival curves of STm after co-incubation with free OMVs at different doses measured by recording the MFI of expressed mCherry. Data are presented as mean values ± SD (*n* = 4, from independent experiments).

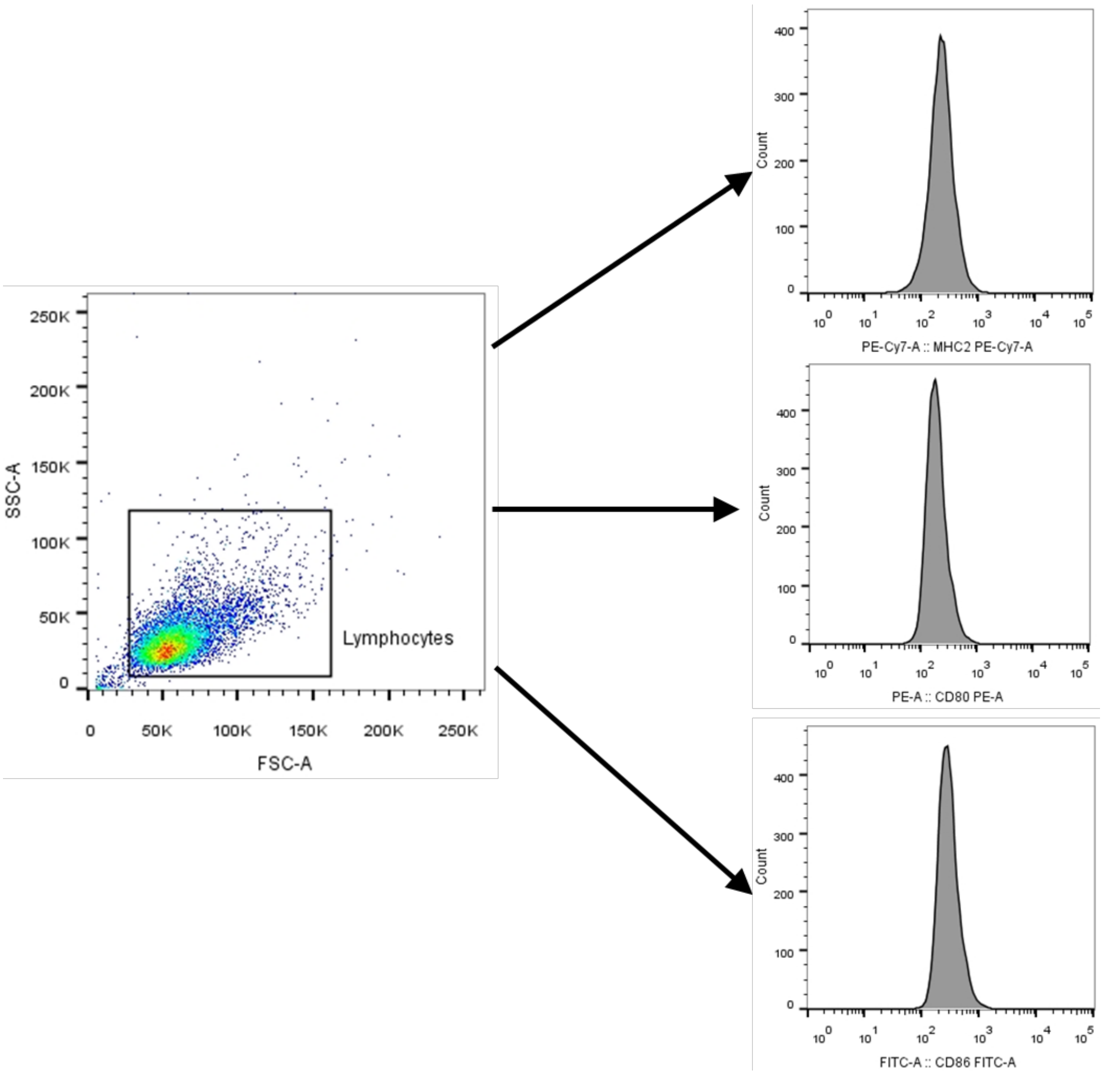

Supplementary Figure 5 Gating strategy to sort MHC II$^+$, CD80$^+$, and CD86$^+$ cells from DC 2.4 cells.

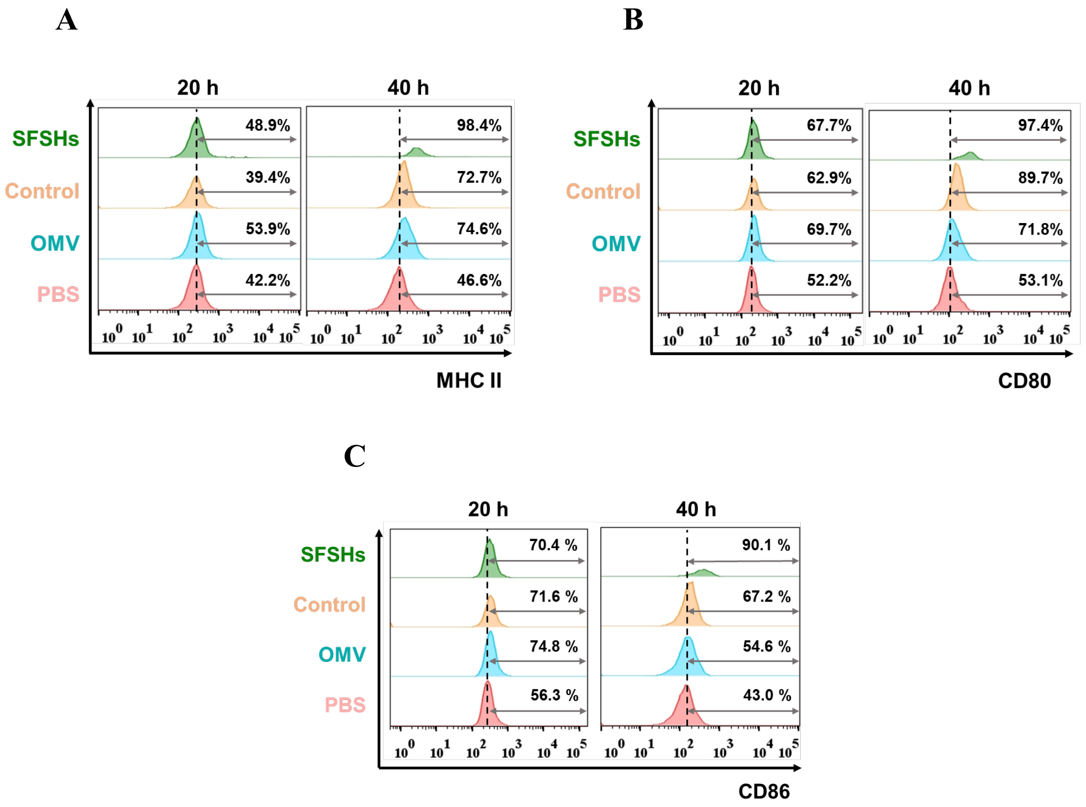

Supplementary Figure 6 FCM histograms of DC2.4 cells after incubation with PBS, free OMVs, control polyacrylamide hydrogel, and SFSHs for 20 h and 40 h, respectively.

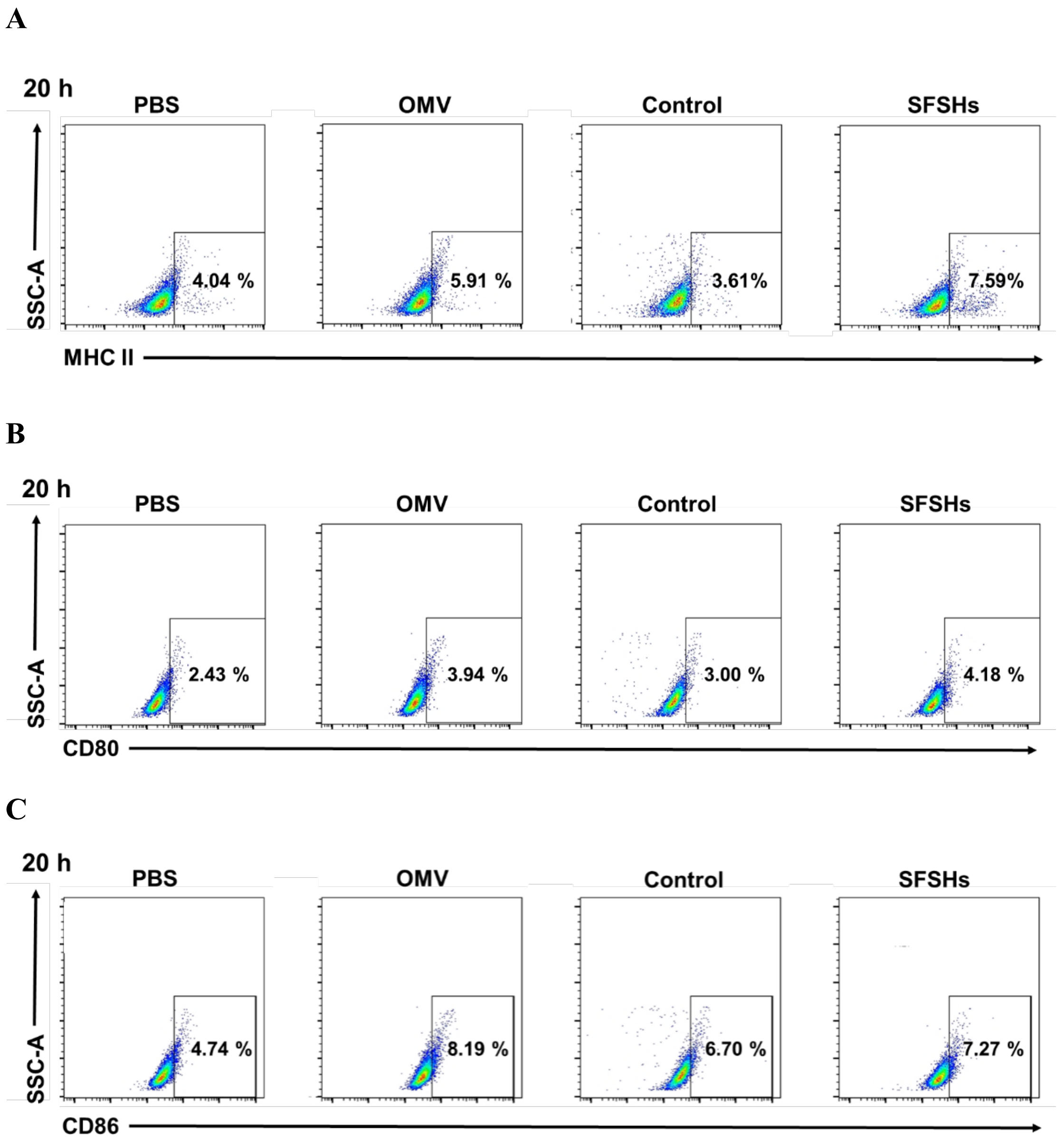

Supplementary Figure 7 FCM scatter plots of expression levels of (**A**) MHC II, (**B**) CD80, and (**C**) CD86 on DC2.4 cells after incubation with PBS, free OMVs, control polyacrylamide hydrogel, and SFSHs for 20 h.

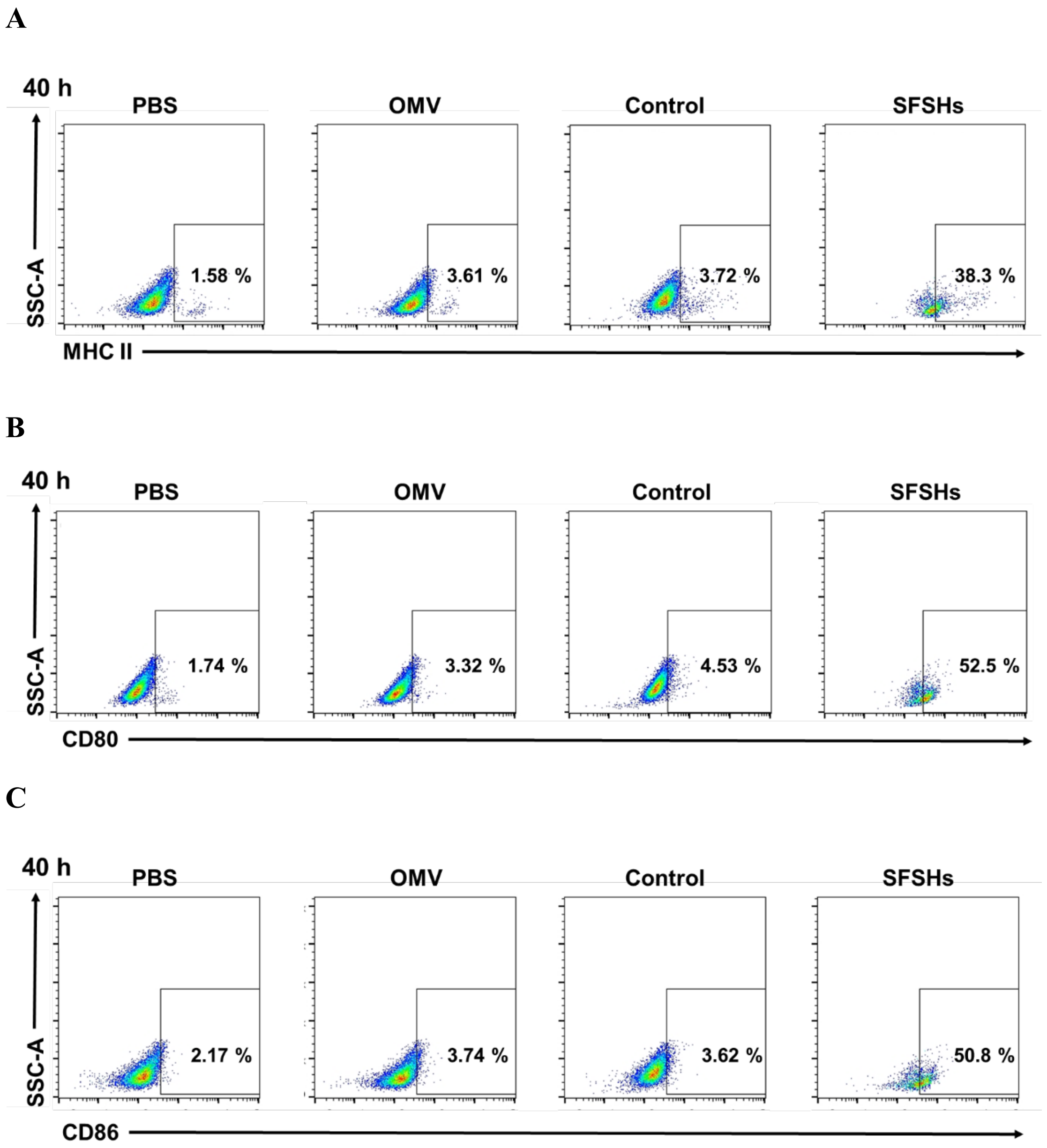

Supplementary Figure 8 FCM scatter plots of expression levels of (A) MHC II, (B) CD80, and (C) CD86 on DC2.4 cells after incubation with PBS, free OMVs, control polyacrylamide hydrogel, and SFSHs for 40 h.

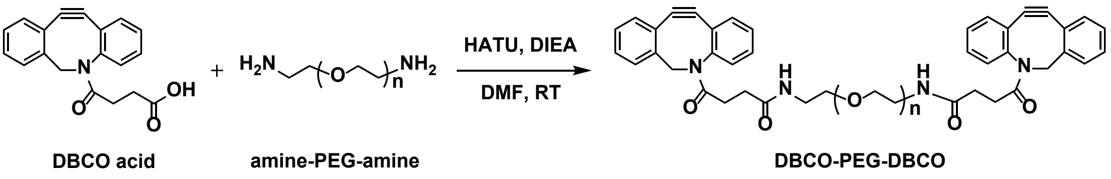

Supplementary Figure 9 Synthetic route of DBCO-PEG-DBCO.

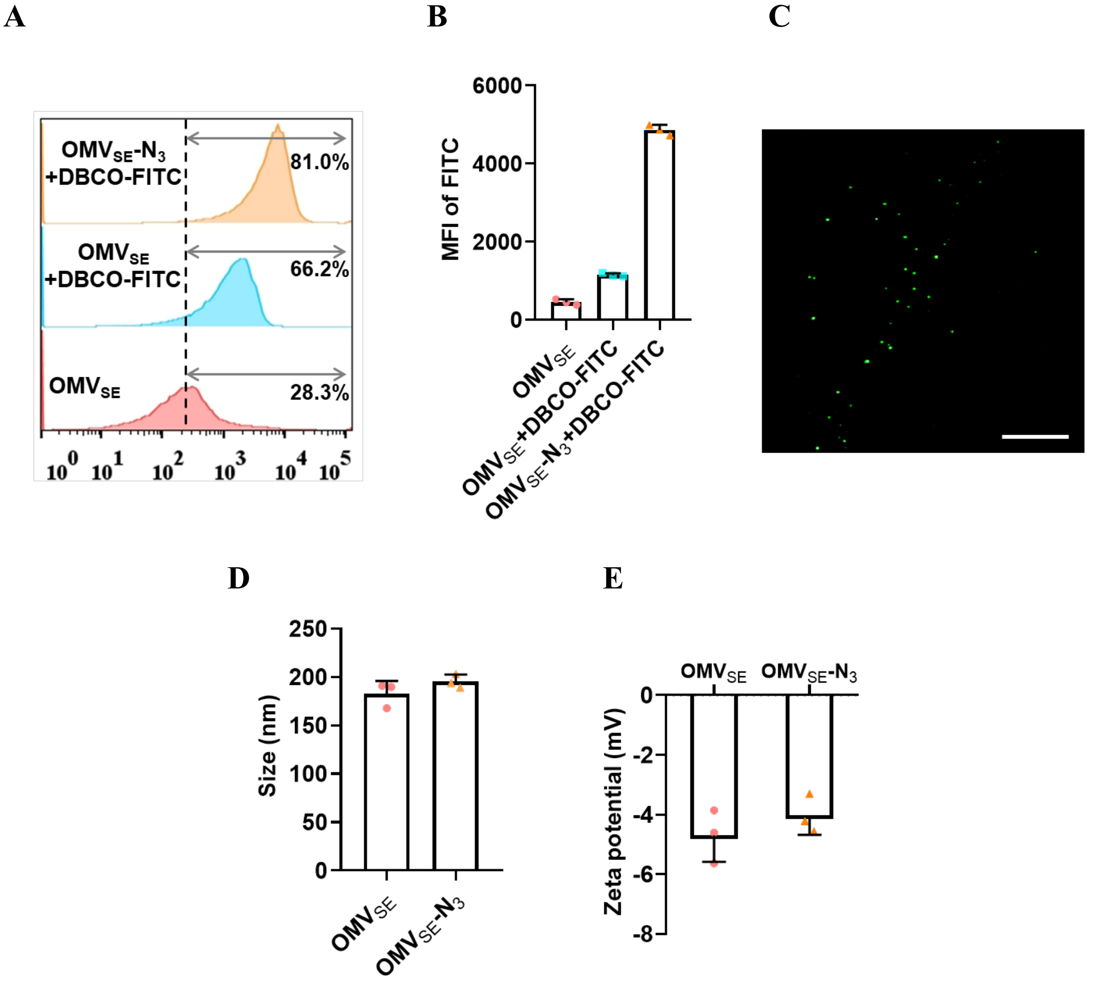

Supplementary Figure 10 (**A**) FCM histograms of OMV_{SE} and OMV_{SE}-N_3 after co-incubation with DBCO-FITC at 25 °C for 0.5 h. (**B**) MFI values of FITC-labeled OMV_{SE} and OMV_{SE}-N_3. (**C**) LSCM imaging of FITC-labeled OMV_{SE}-N_3. Scale bar: 25 μm. (**D**) Average size and (**E**) zeta potential of OMV_{SE} and OMV_{SE}-N_3. Data are presented as mean values ± SD (n = 3, from independent experiments).
